# Where did the attack come from? The mysterious case of paralysis mimicking Guillain-Barré syndrome: Case report

**DOI:** 10.1097/MD.0000000000042118

**Published:** 2025-05-23

**Authors:** Wiktor Wagner, Michał Krawiec, Adam Iwanicki, Paweł Krupa, Barbara Gębka-Kępińska, Mateusz Lubiński, Szymon Białka

**Affiliations:** a Student Scientific Society at the Department of Anaesthesiology and Intensive Therapy in Zabrze, Medical University of Silesia in Katowice, Katowice, Poland; b Department and Clinic of Neurology, Faculty of Medical Sciences in Zabrze, Medical University of Silesia in Katowice, Katowice, Poland; c Department of Anesthesiology and Intensive Therapy, Faculty of Medical Sciences in Zabrze, Medical University of Silesia in Katowice, Katowice, Poland.

**Keywords:** case report, Guillain-Barré syndrome, paralysis, plasmapheresis, tick-borne Encephalitis

## Abstract

**Rationale:**

Tick-borne encephalitis (TBE) can present with neurological symptoms mimicking Guillain-Barré syndrome (GBS), posing a significant diagnostic challenge. Misidentification may lead to delayed or inappropriate treatment, increasing the risk of complications.

**Patient concerns:**

A 49-year-old man presented with flu-like symptoms and progressive paralysis of the left upper limb following a tick bite. His condition rapidly deteriorated despite initial therapy, including facial nerve palsy, further paralysis of the left upper limb, and swallowing difficulties.

**Diagnoses:**

The initial clinical picture, cerebrospinal fluid analysis showing elevated protein, and electromyography suggested GBS.

**Interventions:**

The patient underwent 6 plasmapheresis sessions for suspected GBS, with no neurological improvement.

**Outcomes:**

Due to a lack of improvement and further magnetic resonance imaging findings, a repeated lumbar puncture and serological testing revealed the correct diagnosis of TBE, confirmed by the presence of IgM and IgG antibodies against the TBE virus. After stabilization and intensive care unit discharge, the patient was transferred to a neurology ward and later to a rehabilitation unit. Significant neurological improvement was observed, although partial left upper limb paresis and facial nerve palsy persisted. He was eventually discharged with continued outpatient neurology and cardiology follow-up.

**Lessons:**

This case highlights the importance of detailed medical history, including tick exposure, and maintaining a broad differential diagnosis in acute flaccid paralysis. Early consideration of infectious etiologies, particularly in endemic regions, is essential to prevent unnecessary interventions and improve patient outcomes.

## 1. Introduction

### 1.1. Tick-borne encephalitis

Tick-borne encephalitis (TBE) is a disease of the central nervous system caused by the TBE virus (TBEV), a member of the Flaviviridae family. There are 3 main types of the virus: the European type (TBEV-Eu), the Far Eastern type (TBEV-Fe), and the Siberian type (TBEV-Sib).^[1]^ The natural reservoir of the pathogen consists of small mammals and ticks from the *Ixodes* genus. Arachnids transmit the virus by acquiring it along with the blood of their victims. The disease occurs across almost all of Eurasia, from France to Japan, with exceptionally high incidence in Poland and Czech Republic.^[2]^

The incubation period for TBE typically ranges from 4 to 28 days. Early symptoms appear within 2 to 10 days and are nonspecific and include fever, fatigue, general weakness, headache, and muscle pain. Later stages of the disease are characterized by the following symptoms: high fever, severe headache, and signs of central nervous system involvement – neck stiffness, seizures, paralysis, balance disorders, and altered consciousness.^[3]^

Diagnosing the disease is challenging, mainly due to nonspecific early symptoms. Laboratory tests may reveal mild leukopenia and thrombocytopenia. In the later stages of the disease, elevated white blood cell counts and pleocytosis in cerebrospinal fluid (CSF) can be observed. The diagnosis is ultimately confirmed through tests detecting specific IgG and IgM antibodies against the virus antigens.^[4]^

Currently, there is no casual treatment for TBE. Therapy includes antipyretics, antiemetics, analgesics, proper nutrition, and fluid and electrolyte balance maintenance. The use of anticonvulsants should be considered to prevent further damage to the central nervous system. If muscle paralysis leads to respiratory dysfunction, intubation and mechanical ventilation are initiated.^[5]^ A vaccine against TBE is available, but it is mainly recommended for people at occupational risk of exposure to the ticks that transmit the disease.^[1,4]^

### 1.2. Guillain-Barré syndrome

Guillain-Barré Syndrome (GBS) is a rare, acute, inflammatory autoimmune disorder characterized by demyelinating and axonal motor neuropathy. Its incidence ranges from 0.89 to 1.89 cases per 100,000 people, with a higher prevalence in men than in women with a 3:2 ratio. GBS is often a post-inflammatory condition triggering gastrointestinal and respiratory infections, with *Campylobacter jejuni* being the most identified pathogen.^[6]^ Noninflammatory factors, such as vaccination, surgery, or even snake bites,^[7]^ have also been implicated in some cases.

The first symptom of the disease is rapidly progressing symmetrical paralysis of the limbs. Additional symptoms may include paresthesia, root pain, weakened or absent deep tendon reflexes, and sensory deficits. Diagnosis is based on nerve conduction studies and electromyography. A thorough analysis of each case is crucial, as many conditions (known as “mimics”) present with similar clinical manifestations.^[8–10]^

GBS is treated symptomatically and includes plasmapheresis, which is characteristic of the treatment of certain autoimmune diseases.^[11]^ However, in conditions with a different neurological origin, this method is ineffective and can lead to complications such as hypotension, arrhythmias, or hemolysis.^[12]^

## 2. Case report

A 49-year-old male patient presented to the Primary care Physician with the symptoms of sore throat, weakness, low-grade fever, muscle aches, and general malaise. The patient reported a tick bite 9 days before the visit, which he had removed himself using tweezers. Despite being prescribed antibiotic therapy, his condition did not improve, and paralysis of the left arm had developed. He was referred to the neurology department for further evaluation. During the neurological examination, the patient was conscious and oriented both auto and allopsychically. Upon examination, additional findings included central paresis of the left VII cranial nerve, anisocoria with the left pupil more significant than the right, flaccid paralysis of the left upper limb with areflexia (triceps reflex on the left side±), and hyperesthesia of the left upper limb. Ataxia of the left upper limb could not be assessed. The lower limbs showed no paresis, ataxia, or superficial or profound sensation disturbances. The Babinski sign was bilaterally absent. Initial diagnostic tests were conducted: head computer tomography (CT) scan revealed no abnormalities. Cervical spine magnetic resonance imaging (MRI) showed spondylosis and idiopathic changes in the cervical spine with C6/C7 spinal canal stenosis; electromyography illustrated features of axonal polyneuropathy of a motor type, general analysis of cerebrospinal fluid: CSF was clear and colorless with no leukocytes present. The culture for aerobic and anaerobic bacteria was negative. Tests for autoantibodies were not performed.

Based on the clinical presentation, initial findings, and results of CSF analysis, Guillain-Barré syndrome was diagnosed. The possible diagnosis of tick-borne encephalitis (due to tick bite reported in medical history) wasn’t considered, due to low tick activity in autumn. What is more, imaging techniques haven’t revealed any abnormalities in brain structures and peripheral symptoms strongly resembled those in GBS. The patient was admitted to the intensive care unit (ICU) of the Independent Public Clinical Hospital No. 1, Prof Stanisław Szyszko Silesian Medical University in Katowice for therapeutic plasmapheresis.

Upon admission to the ICU, the patient presented with symptoms of flaccid paralysis in the left upper limb, retaining slight movement in the fingers. He also displayed a drooping left corner of the mouth and difficulty swallowing liquid foods. Additionally, neck stiffness at 1cm width was recorded, and narrow pupil dilation was observed. The pulmonary fields showed decreased vesicular breath sounds bilaterally at the lung bases, with crackles in the same regions. The patient was hemodynamically stable, with blood pressure of 120/80 mm Hg, heart rate of 70 bpm, and no peripheral edema. The abdomen was soft and non-tender with no pathological resistance and audible peristalsis. The patient was catheterized, with observed straw-yellow urine in the collection bag.

The patient’s vital signs were continuously monitored in the ICU, vascular cannulation was performed, and laboratory and microbiological samples were taken. Passive oxygen therapy was continued, along with broad-spectrum empirical antibiotic therapy that was initiated and adjusted based on microbiological results, antiviral medications, analgesics, mucolytics, prokinetics, antiarrhythmic and antihypertensive medications, gastric mucosal protectants, steroids, diuretics, probiotics, thromboembolic prophylaxis, neuroprotective drugs, and electrolyte imbalances were corrected. After catheter insertion, the patient underwent 6 therapeutic plasmapheresis sessions, filtering 22,300 mL of plasma. The patient did not have complications during the procedures, but no neurological improvement was observed after completion. During hospitalization, the swallowing difficulties worsened, and on the second day, a nasogastric tube was inserted to provide enteral feeding and hydration. On the third day, due to increasing respiratory distress, CO_2_ accumulation, and respiratory acidosis obtained from the arterial blood gases, the patient was intubated, and mechanical ventilation was initiated. Along with sedation and intensive shock, treatment for hypotension was provided. Due to atelectasis, the patient underwent several bronchofiberoscopic procedures to clean the bronchial tree. On the 4th day of hospitalization, anterior neck swelling was observed, which dominated on the right side. The patient underwent a repeat CT scan of the head and neck with contrast, which showed free fluid accumulating between the muscles of the right side of the neck, causing a bulging outline of the neck. Still, no contrast medium was seen extravasating the outside of the vessel. The central venous catheter was removed from the right internal jugular vein, resulting in a local improvement. As a result of the multidirectional treatment applied, respiratory function improved. On the 11th day, the noradrenaline infusion was discontinued due to the stabilization of blood pressure. However, neurological examination on the 12th day of treatment revealed worsening symptoms of progressing flaccid paralysis of the left upper limb with preserved trace movement in the fingers, and further muscle strength weakness in the lower limbs, rated at 2 to 3 points on Lovett scale. Neck stiffness increased to 2.5 fingers in width; the Kerning sign is positive bilaterally, while Babinski and Lasègue signs were negative without clonus.

**Figure 1.**
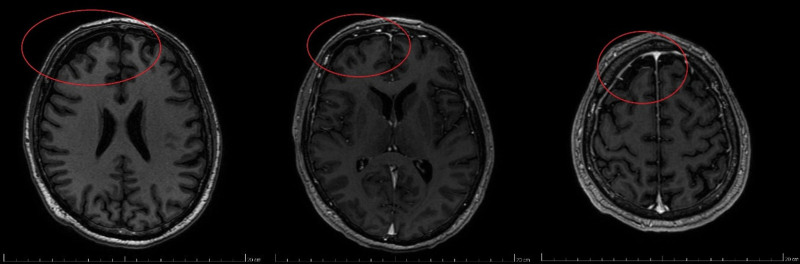
MRI of the patient’s head, performed on the 12th day of hospitalization. Segmental enlargement of the perimesencephalic fluid spaces is visible near the right frontal lobe, as well as post-contrast enhancement of the dura mater, slightly thickened in the right frontal area. MRI = magnetic resonance imaging.

Due to the lack of improvement with an overall worsening neurological condition and the fact that previously described changes in the cervical spinal cord MRI were not specific to Guillain-Barré syndrome, on the same day, a repeat MRI of the head and cervical spine with contrast was performed. Compared to the previous examination, this showed segmental enlargement of the peri mesencephalic fluid spaces near the right frontal lobe and post-contrast enhancement of the dura mater, slightly thickened in the right frontal area (Fig. 1). The MRI report also confirmed degenerative changes in the cervical spine at C5 to C7 levels, with spinal canal stenosis and right neural foramen stenosis at C6/C7. As a result, according to the recommendation of the consulting neurologist, on the 14th day of treatment, a repeat lumbar puncture was performed to collect CSF for general and microbiological analysis. Abnormal results included protein level: 1177.05 mg/L (standard: 150–450 mg/L) and pleocytosis with mononuclear cells with T-lymphocyte morphology: 0.021 × 10^3^/μL (normal: 0–0.005/μL). Additionally, the patient’s blood was sent to the Provincial Sanitary and Epidemiological Station in Katowice to test antibodies in IgG and IgM classes against TBEV (the virus causing tick-borne encephalitis), confirming the presence of both classes’ antibodies. Due to persistent swallowing difficulties and a weak cough reflex, with the patient’s consent, a tracheotomy was performed on October 18, 2022. The following day, mechanical ventilation support was discontinued, and passive oxygen therapy was applied through the tracheotomy thereafter. Based on most recent MRI scans with present encephalitis, clinical presentation and antibodies tests results, TBEV diagnosis was confirmed.

The patient is conscious, responsive, and following commands, with pupils reacting to light. He presents with peripheral facial nerve palsy, swallowing difficulties, severe weakness of the left upper limb (with a trace of thumb movement), and spontaneous movement of the other limbs with weakened muscle strength. Neck stiffness was noted at a 2-finger distance, breathing spontaneously through a tracheotomy with passive oxygen therapy at a flow of 2 L/min, hemodynamically stable, fed with an industrial diet, and hydrated via a nasogastric tube. The patient was afebrile and transferred to the Neurology Department of the WSS No. 5 in Sosnowiec for further treatment.

Upon admission to the Neurology Department, the patient was conscious, responsive, and following commands but unable to speak. Respiratory function was stable, exhibiting peripheral left-sided VII cranial nerve palsy, neck stiffness to a 2-finger distance, a positive Kernig sign, flaccid paralysis of the left upper limb with areflexia, partial paralysis of the lower limbs (graded 3/4 on the Lovett scale), and absent bilateral Babinski sign.

On the first day of hospitalization, the patient developed resting dyspnea, decreased oxygen saturation, and a significant increase in D-dimer levels. An urgent echocardiogram revealed signs of pulmonary embolism with right heart enlargement and overload (RV-37 mm) and significant tricuspid regurgitation. A chest CT angiogram confirmed a massive bilateral pulmonary embolism. Following a cardiology consultation, anticoagulant treatment was started with unfractionated heparin infusion under activated partial thromboplastin time control. However, due to a sharp drop in platelet count (30,000), unfractionated heparin was abandoned, and rivaroxaban was introduced. After treatment, dyspnea resolved, and D-dimer levels normalized.

During a 23-day hospitalization, the patient’s condition improved enough for transfer to the Neurological Rehabilitation Department, where, after 52 days of rehabilitation, he was referred to a follow-up to the cardiology and neurology outpatient clinics. During the follow-up visit, partial weakness of the left upper limb and facial nerve palsy persisted despite rehabilitation.

## 3. Discussion

Tick-borne encephalitis is becoming an increasingly serious health issue worldwide, particularly in Eastern Europe. In Poland, the number of tick-borne diseases has been rising year by year, posing a significant challenge for primary healthcare providers and neurology specialists.^[1,13]^

Tick-borne encephalitis presents a particular risk to patients due to its insidious onset and initially nonspecific symptoms.^[14]^ Neurological symptoms occurring in the early stages of the disease, such as rapidly progressing flaccid paralysis, diminished reflexes, facial nerve damage, or swallowing disorders, can lead to misdiagnosis, as these symptoms resemble other conditions, including Guillain-Barré syndrome.^[15]^ Misdiagnosing the disease may result in ineffective and potentially harmful treatments, significantly worsening the patient’s prognosis.^[16]^ This underscores the importance of obtaining a thorough and detailed medical history, including a potential tick bite and performing diagnostic tests for Lyme disease, the most common tick-borne disease – and other tick-borne illnesses.^[17,18]^

The hospitalization of our patient emphasizes how severe tick-borne encephalitis can be, potentially leading to life-threatening conditions. It is also noticeable how severe complications can arise, significantly impair the quality of life as they appear, even after the patient is discharged from the Intensive Care Unit and Neurology Department.

The clinical case described above, with the initial misdiagnosis of Guillain-Barré syndrome, highlights the crucial importance of detailed medical history and the comprehensive analysis of the broad range of potential etiologies for the patient’s symptoms. It emphasizes the fundamental value of the medical interview in the face of modern diagnostic methods.^[19]^

## Author contributions

**Conceptualization:** Wiktor Wagner, Adam Iwanicki, Paweł Krupa, Szymon Białka.

**Data curation:** Wiktor Wagner, Michał Krawiec, Adam Iwanicki, Paweł Krupa, Szymon Białka.

**Investigation:** Wiktor Wagner, Paweł Krupa, Adam Iwanicki, Michał Krawiec.

**Methodology:** Wiktor Wagner.

**Project administration:** Wiktor Wagner, Szymon Białka.

**Resources:** Szymon Białka, Barbara Gębka-Kępinska,Mateusz Lubinski

**Supervision:** Barbara Gębka-Kępińska, Mateusz Lubiński, Szymon Białka.

**Validation:** Wiktor Wagner, Barbara Gębka-Kępińska, Mateusz Lubiński, Szymon Białka.

**Visualization:** Wiktor Wagner.

**Writing – original draft:** Wiktor Wagner, Michał Krawiec, Adam Iwanicki.

**Writing – review & editing:** Barbara Gębka-Kępińska, Mateusz Lubiński, Szymon Białka.
